# PLUS-IS-LESS project: Procalcitonin and Lung UltraSonography-based antibiotherapy in patients with Lower rESpiratory tract infection in Swiss Emergency Departments: study protocol for a pragmatic stepped-wedge cluster-randomized trial

**DOI:** 10.1186/s13063-023-07795-y

**Published:** 2024-01-25

**Authors:** Cécile Bessat, Roland Bingisser, Markus Schwendinger, Tim Bulaty, Yvan Fournier, Vincent Della Santa, Magali Pfeil, Dominique Schwab, Jörg D. Leuppi, Nicolas Geigy, Stephan Steuer, Friedemann Roos, Michael Christ, Adriana Sirova, Tanguy Espejo, Henk Riedel, Alexandra Atzl, Fabian Napieralski, Joachim Marti, Giulio Cisco, Rose-Anna Foley, Melinée Schindler, Mary-Anne Hartley, Aurélie Fayet, Elena Garcia, Isabella Locatelli, Werner C. Albrich, Olivier Hugli, Noémie Boillat-Blanco, for the PLUS-IS-LESS study group, Noémie Boillat-Blanco, Werner C. Albrich, Nicolas Garin, Björn Mattsson, Stephan Gasser, Dieter Von Ow

**Affiliations:** 1 Infectious Diseases Service, University Hospital of Lausanne and University of Lausanne, Lausanne, Switzerland; 2 Emergency Department, University Hospital of Basel, Basel, Switzerland; 3 Emergency Department, Cantonal Hospital of Baden, Baden, Switzerland; 4 Emergency Department, Intercantonal Hospital of Broye, Payerne, Switzerland; 5 Emergency Department, Hospital of Neuchâtel, Neuchâtel, Switzerland; 6 Emergency Department, Hospital Riviera-Chablais, Rennaz, Switzerland; 7 Emergency Department and University Medicine, Cantonal Hospital Baselland, Liestal, Switzerland; 8 Emergency Department, St Claraspital, Basel, Switzerland; 9 Emergency Department, Cantonal Hospital of Lucerne, Lucerne, Switzerland; 10 Emergency Department, Cantonal Hospital of St Gallen, St Gallen, Switzerland; 11 Health Economics and Policy Unit, Department of Epidemiology and Health Systems, Centre for Primary Care and Public Health (Unisanté), University of Lausanne, Lausanne, Switzerland; 12 Qualitative research platform, social sciences sector, Department of Epidemiology and Health Services, Centre for Primary Care and Public Health (Unisanté), University of Lausanne, Lausanne, Switzerland; 13 School of Health Sciences HESAV, University of Applied sciences of Western Switzerland, HES-SO, Lausanne, Switzerland; 14 Intelligent Global Health Research Group, Machine Learning and Optimization Laboratory, Swiss Federal Institute of Technology (EPFL), Lausanne, Switzerland; 15 Clinical Research Center (CRC), University Hospital of Lausanne and University of Lausanne, Lausanne, Switzerland; 16 Emergency Department, University Hospital of Lausanne and University of Lausanne, Lausanne, Switzerland; 17 Division of Infectious Diseases & Hospital Epidemiology, Cantonal Hospital St Gallen, St Gallen, Switzerland

**Keywords:** Lower respiratory tract infection, Community-acquired pneumonia, Lung ultrasound, Procalcitonin, Antibiotic prescription, Algorithm, Diagnostic tool, Emergency department, Clinical trial, Protocol

## Abstract

**Background:**

Lower respiratory tract infections (LRTIs) are among the most frequent infections and a significant contributor to inappropriate antibiotic prescription. Currently, no single diagnostic tool can reliably identify bacterial pneumonia. We thus evaluate a multimodal approach based on a clinical score, lung ultrasound (LUS), and the inflammatory biomarker, procalcitonin (PCT) to guide prescription of antibiotics. LUS outperforms chest X-ray in the identification of pneumonia, while PCT is known to be elevated in bacterial and/or severe infections. We propose a trial to test their synergistic potential in reducing antibiotic prescription while preserving patient safety in emergency departments (ED).

**Methods:**

The PLUS-IS-LESS study is a pragmatic, stepped-wedge cluster-randomized, clinical trial conducted in 10 Swiss EDs. It assesses the PLUS algorithm, which combines a clinical prediction score, LUS, PCT, and a clinical severity score to guide antibiotics among adults with LRTIs, compared with usual care. The co-primary endpoints are the proportion of patients prescribed antibiotics and the proportion of patients with clinical failure by day 28. Secondary endpoints include measurement of change in quality of life, length of hospital stay, antibiotic-related side effects, barriers and facilitators to the implementation of the algorithm, cost-effectiveness of the intervention, and identification of patterns of pneumonia in LUS using machine learning.

**Discussion:**

The PLUS algorithm aims to optimize prescription of antibiotics through improved diagnostic performance and maximization of physician adherence, while ensuring safety. It is based on previously validated tests and does therefore not expose participants to unforeseeable risks. Cluster randomization prevents cross-contamination between study groups, as physicians are not exposed to the intervention during or before the control period. The stepped-wedge implementation of the intervention allows effect calculation from both between- and within-cluster comparisons, which enhances statistical power and allows smaller sample size than a parallel cluster design. Moreover, it enables the training of all centers for the intervention, simplifying implementation if the results prove successful.

The PLUS algorithm has the potential to improve the identification of LRTIs that would benefit from antibiotics. When scaled, the expected reduction in the proportion of antibiotics prescribed has the potential to not only decrease side effects and costs but also mitigate antibiotic resistance.

**Trial registration:**

This study was registered on July 19, 2022, on the ClinicalTrials.gov registry using reference number: NCT05463406.

**Trial status:**

Recruitment started on December 5, 2022, and will be completed on November 3, 2024. Current protocol version is version 3.0, dated April 3, 2023.

**Supplementary Information:**

The online version contains supplementary material available at 10.1186/s13063-023-07795-y.

## Introduction

### Background and rationale

Community-acquired lower respiratory tract infections (LRTI) are one of the most common motivations for consultations in the emergency department (ED) and stand as the leading cause of inappropriate antibiotic prescription [1]. LRTIs span a wide range of diseases, from self-limited acute bronchitis and infectious exacerbations of chronic obstructive pulmonary disease (COPD) to life-threatening pneumonia. Viruses cause the majority of LRTIs and are also identified in a quarter of community-acquired pneumonia (CAP), with an even higher prevalence during the SARS-CoV-2 outbreak [2, 3].

When assessing patients with LRTIs, the challenge for ED physicians is to identify those with bacterial CAP, who are most likely to benefit from antibiotics. The performance of current tools to diagnose CAP in patients with LRTI is limited. Chest X-ray, the current reference standard for pneumonia diagnosis, has poor accuracy [4, 5]: based on clinical features and chest X-ray, pneumonia is largely overestimated, as a third of patients have a normal CT scan [6]. The low diagnostic accuracy of existing tools is one of the causes of inappropriate antibiotic prescriptions and excessive utilization of costly resources (blood tests, radiology, and microbiology) among patients with LRTIs [7–11]. Although 40% of patients with LRTIs have CAP in the ED, 60 to 80% of patients and almost all those with CAP receive antibiotics [3, 12, 13]. Besides their side effects, antibiotic overuse alters the microbiome and selects for antibiotic resistance [14, 15].

Several diagnostic tests can assist in identifying patients with LRTI which require antibiotics. Lung ultrasound (LUS) can be performed quickly at the bedside without radiation and has a better diagnostic performance than chest X-ray to detect infiltrates. Recent meta-analyses have shown that LUS has an excellent sensitivity (92–94%) and specificity (74–96%) in diagnosing CAP in adults ED patients using chest CT as gold standard [16–18]. However, based on a recent review, 25% of pneumonias are viral and cannot—based on imaging alone—be distinguished from a bacterial pneumonia [2]. Procalcitonin (PCT) is a host inflammatory biomarker which is usually elevated in bacterial and/or severe infections [19]. While PCT can be used to safely guide antibiotic use, its impact on their prescription remains controversial mainly due to variable physician adherence to PCT guidance [13, 20, 21]. If none of these tools in isolation is sufficient to optimize antibiotic prescription, a combined approach could improve diagnostic performance, ensure patients’ safety, and maximize clinicians’ adherence to guidance.

To overcome the limited performance of guideline-recommended diagnostic approach and the shortcomings of previously tested interventions for the management of LRTIs, we provide ED physicians with a novel simple clinical management algorithm: the PLUS algorithm. The *PLUS* algorithm integrates a clinical predictive score for CAP (Van Vugt score), *LUS*, *P*CT, and, in case of discordant results between lung ultrasound and PCT, a clinical severity score (DS-CRB-65). The purpose of this algorithm is to improve the identification of patients with CAP requiring antibiotics and decrease unnecessary prescriptions in adult patients with LRTIs managed in Swiss EDs. It guides the bedside decision-making for antibiotic prescription and helps physicians to safely withhold unnecessary prescriptions in adult patients with LRTIs.

### Objectives

The primary safety objective of the Procalcitonin and Lung UltraSonography based antibiotherapy in patients with Lower rESpiratory tract infection in Swiss Emergency Departments (PLUS-IS-LESS) study is to demonstrate a non-inferiority of the PLUS algorithm, in terms of clinical failure by day 28 when compared with usual care, among patients with LRTIs in the ED. The co-primary efficacy objective is to show a 15% reduction in the proportion of patients with LRTIs prescribed an antibiotic by day 28 in the intervention group compared to the usual care group.

Secondary objectives of the study are to compare between the intervention and control groups the quality of life (the inconvenient nature of CAP-related symptoms) on day 7, day 28, and day 90, the duration of ED stay, the rate and duration of hospitalization, the proportion of patients prescribed an antibiotic for a new respiratory infection between day 28 and 90, as well as the proportion of patient with antibiotic-related side effects and *Clostridioides difficile* infection. In addition, we will evaluate the acceptability and feasibility of the intervention through identification of barriers and facilitators for patients and physicians, compare quality-adjusted life years (QALYs), derived from responses to the EQ-5D-5L questionnaire (euroqol.org), assess the incremental cost-effectiveness of the intervention as compared to usual care, develop advanced automatic LUS image analysis method using machine learning to assist in LUS diagnosis and risk stratification, assess the proportion of physicians reaching proficiency in LUS image/video acquisition and interpretation after the training module, and evaluate the diagnostic performance of physician “gestalt” and Van Vugt prediction score for CAP diagnosis. 

## Methods: Study design, participants, interventions, and outcomes

### Trial design

The PLUS-IS-LESS study is a pragmatic, investigator-initiated, stepped-wedge cluster-randomized, controlled clinical trial investigating the PLUS algorithm to guide prescription of antibiotics among adults ED patients with LRTI. This protocol is written based on the SPIRIT checklist (Additional file 1) [22].

### Study setting

The study takes place in 10 Swiss EDs of 9 centers: 6 tertiary hospitals (Cantonal Hospital of Baden, Cantonal Hospital Baselland, Cantonal Hospital of Luzern, Cantonal Hospital of St. Gallen, University Hospital of Basel, University Hospital of Lausanne) and 4 regional hospitals (Hospital of Neuchâtel, Intercantonal Hospital of Broye, Hospital Riviera-Chablais and St. Claraspital Basel). Cantonal Hospital Baselland and St. Claraspital Basel represent one study center as they share guidelines and medical practices. Baseline characteristics of the 10 participating study hospitals are presented in Table 1.

**Table 1:** Baseline characteristics of the 10 participating study hospitals in Switzerland

	Center 1	Center 2	Center 3	Center 4	Center 5	Center 6	Center 7	Center 8	Center 9	
	Cantonal Hospital of St Gallen	University Hospital of Lausanne (2021)	Hospital of Neuchâtel (2021)	University Hospital of Basel	Cantonal Hospital of Lucerne	Intercantonal Hospital of Broye	Hospital Riviera-Chablais	Cantonal Hospital of Baden	Cantonal Hospital Baselland	St Claraspital Basel
Nr. of adults acute-care beds 2022	710	814	296	700	630	100	264	389	160	230
Nr. of visits in the emergency department in 2022	48,839	43,509	32,172	56,800	25,794	18,158	35,206	65,000	29,697	16,769

### Eligibility and exclusion criteria

All adult patients, aged 18 or older, presenting with an acute LRTI, are screened for inclusion by study nurses (SN) during working hours (weekdays 8am–5pm). Acute LRTI is defined, according to the European Society of Clinical Microbiology and Infectious Disease (ESCMID) guidelines [4], as an acute illness of less than 21 days, featuring at least one lower respiratory tract symptom, i.e., cough, sputum, dyspnea, or chest pain with no alternative explanation. Every consecutive patient meeting the inclusion and exclusion criteria detailed in Table 2 and providing a signed informed consent is included. SNs and/or physicians are responsible for collecting written patient’s informed consent for each participant.

**Table 2:** Inclusion and exclusion criteria

Inclusion criteria	Exclusion criteria	
Patients aged 18 years or more	Previous receipt of a quinolone, macrolide, or ceftriaxone or more than one dose of any other antibiotic within 72 h prior to enrolment (excepted prophylactic antibiotics or antibiotics given for urinary tract infection)	
Acute LRTI (acute illness, less than 21 days, with at least one lower respiratory tract symptom, i.e., cough, sputum, dyspnea, chest pain, and no alternative explanation)	Acute care hospital stay in the previous 14 days	
At least one of the following clinical criteria:	Cystic fibrosis	
o Focal abnormal auscultation (decreased breath sounds, crackles, bronchial breath sounds)	Severe COPD (≥GOLD 3 or if not available, as a proxy: exacerbation treated with antibiotics during the last 6 months)	
o Fever (documented temperature ≥ 38°C in the last 24 h, including self-measured temperature ≥ 38°C)	Severe immunodeficiency (drug-induced neutropenia with <500 neutrophils/mm^3^, HIV infection with CD4<200 cells/mm^3^, solid organ or bone marrow transplant recipient, prednisone ≥ 20 mg/day for >28 days)	
o Tachypnoea (respiratory rate ≥ 24/min)	Initial admission of the patient in the intensive care unit	
o Tachycardia (heart rate ≥ 100/min	Microbiologically documented infection with SARS-CoV-2 (in the last 10 days)	
	Lack of decision-making capacity	

### Study intervention

#### The PLUS clinical management algorithm

**Fig. 1:**
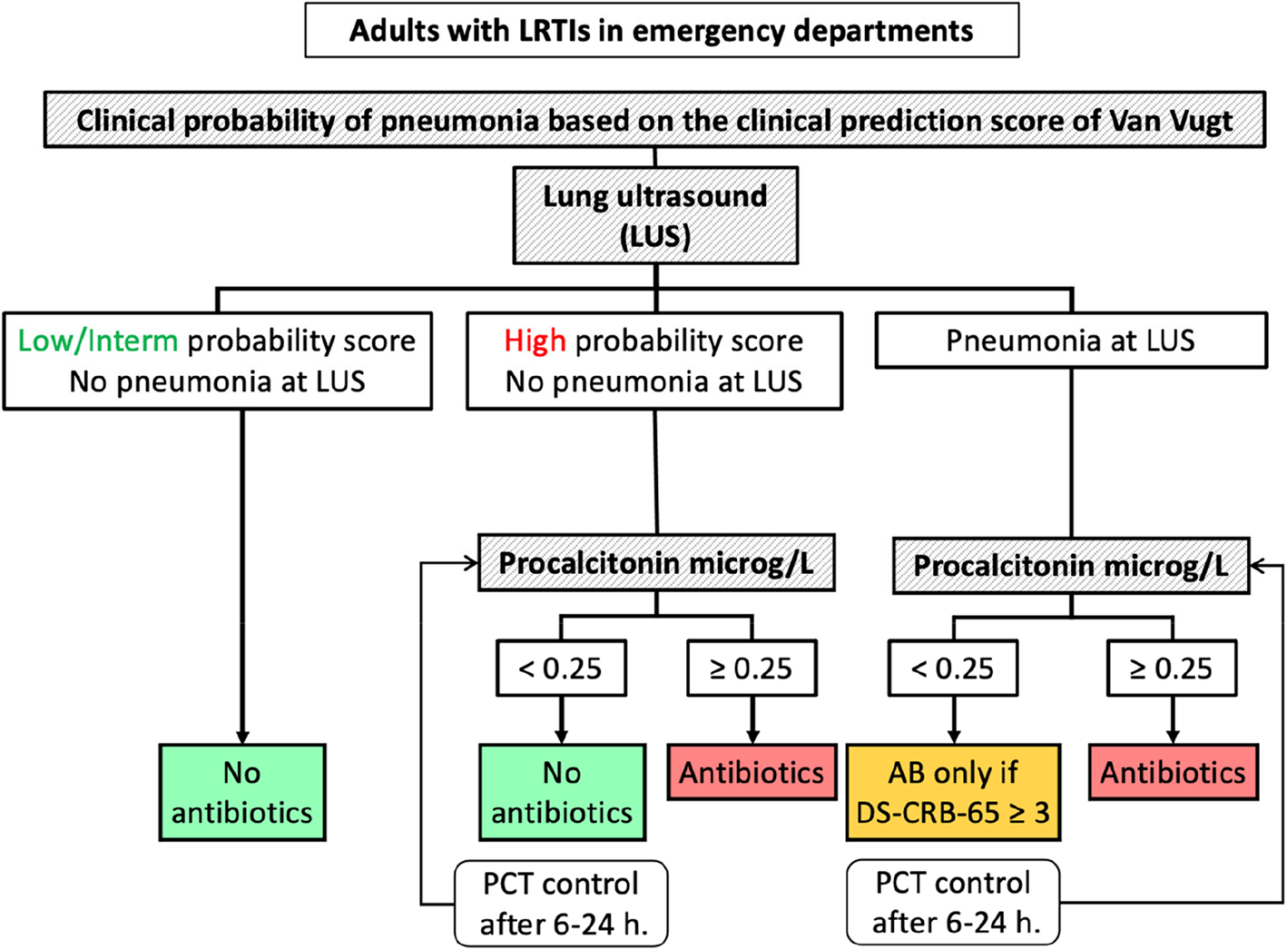
The PLUS algorithm. AB, antibiotics; DS-CRB-65, clinical severity score: add 1 point for disease (D): presence of at least one of the following: congestive heart failure, cerebrovascular disease, chronic renal disease, chronic liver disease or active malignancy; oxygen saturation (S) < 90% to the CRB-65 score, Van Vugt score: 1 point per item: absence of coryza, presence of dyspnea, crackles, and diminished breath sound on auscultation, fever ≥37.8°C, tachycardia ≥100/min; low/intermediate for a score ≤ 2 points and high for a score ≥3 points; PCT, procalcitonin

EDs having switched to the intervention period will manage their patients using the PLUS algorithm (intervention group). The PLUS algorithm is detailed in Fig. 1 and Table 3 following the Template for Intervention Description and Replication (TIDieR) criteria [23]. The algorithm starts with a *validated pneumonia clinical prediction score* (Van Vugt score) [24, 25] that standardizes CAP pre-test probability. The Van Vugt score attributes a point to each of the following items: absence of coryza, presence of dyspnea, crackles, and diminished breath sound on auscultation, fever ≥37.8°C, and tachycardia ≥100/min. The probability of pneumonia is categorized into low/intermediate for a score ≤ 2 points and high for a score ≥3 points.

**Table 3:** Description of the intervention and usual care groups, according to the Template for Intervention Description and replication (TIDieR) criteria [23]

TIDieR criterion	Intervention	Usual care
Item 1. Brief name: Provide the name or a phrase that describes the intervention	PLUS-algorithm	Usual ED patient care
Item 2. Why: Describe any rationale, theory or goal of the elements essential to the intervention	LRTIs are a common motivation for ED consultations and stand as the leading cause of inappropriate antibiotic prescription. LUS has an excellent sensitivity and specificity to identify pneumonia and PCT tends to be higher in bacterial and/or severe infections. None of these tools alone is sufficient to optimize antibiotic prescription, while a combined approach could improve diagnostic performance, ensure safety and better guide clinicians.	Usual care represents real life clinical management.
Item 3. What (materials): Describe any physical or informational materials used in the intervention, including those provided to participants or used in intervention delivery or in training of intervention providers	Participants will receive the informed consent form, with detailed information of the project objectives, procedure, risks and benefits.	Participants will receive the informed consent form, with detailed information of the project objectives, procedure, risks and benefits.
Item 4. What (procedures): Describe each of the procedures, activities or processes used in the intervention, including any enabling or support activities	Participants are managed by the physician in charge with the PLUS-algorithm. A pneumonia clinical prediction score (Van Vugt) is calculated automatically by the algorithm in the eCRF, physicians perform a LUS. In case of pneumonia on LUS and/or elevated clinical prediction score, PCT is measured. The algorithm recommends antibiotics in case of elevated PCT (≥ 0.25 μg/L). In patients with features of pneumonia on LUS and a low PCT, severity score (DS-CRB-65) is calculated. The algorithm recommends antibiotics in patients with a high severity score.	Participants are managed as usual. The physicians decide on antibiotic prescription as they usually do (usually based on a chest X-ray and blood tests)
Item 5. Who provided: For each category of intervention provider (for example, psychologist, nursing assistant), describe their expertise, background and any specific training given	Physicians in charge in the ED, have received a one-hour presentation on the scientific rationale behind the algorithm, a two-hour e-learning training on LUS, and a one-hour theoretical and two-hour practical training by an experienced instructor in LUS	Physician in charge in the ED have received a one-hour training on epidemiology and management of CAP in Switzerland based on Swiss guidelines [26] as well as explanations on the background of the study
Item 6. How: Describe the modes of delivery (such as face-to-face or by some other mechanism, such as internet or telephone) of the intervention and whether it was provided individually or in a group	Face-to-face individual meeting	Face-to-face individual meeting
Item 7. Where: Describe the types of location where the intervention occurred, including any necessary infrastructure or relevant features	ED	ED
Item 8. When and how much: Describe the number of times the intervention was delivered and over what period of time, including the number of sessions, their schedule, and their duration, intensity or dose	The intervention is delivered once during ED management. PCT will be repeated after 6 to 24 hours in a subgroup of patient (all admitted patients who did not receive antibiotics because of a low PCT value and in those in whom the algorithm was overruled). In case of worsening disease within 28 days of enrolment in patients who did not receive antibiotics, reassessment will be done, and PCT will also be repeated.	Once during ED management
Item 9. Tailoring: If the intervention was planned to be personalized, titrated or adapted, then describe what, why, when and how	Not applicable	Not applicable
Item 10. Modifications: If the intervention was modified during the course of the study, describe the changes (what, why, when, how)	Not applicable	Not applicable
Item 11. How well (planned): If intervention adherence or fidelity was assessed, describe how and by whom; if any strategies were used to maintain or improve fidelity, describe them	The study monitoring plan includes the evaluation of study center compliance to the protocol and, non-adhesion to the algorithm will be escalated to the sponsor as a study deviation. Non-adhesion to the algorithm AB recommendation also triggers automatic alerts to the sponsor for direct feedback to the physician in charge.	Not applicable
Item 12: How well (actual): If intervention adherence or fidelity was assessed, describe the extent to which the intervention was delivered as planned	We will describe if the intervention was delivered as planned. Patients with incomplete intervention on day 0 will only be analyzed in the intention-to-treat analysis.	Not applicable

*AB* antibiotic, *CAP* community-acquired pneumonia, *eCRF* electronic case report form, *ED* emergency department, *LRTI* lower respiratory tract infection, *LUS* lung ultrasound, *PCT* procalcitonin, *TIDieR* Template for Intervention Description and replication

**Fig. 2:**
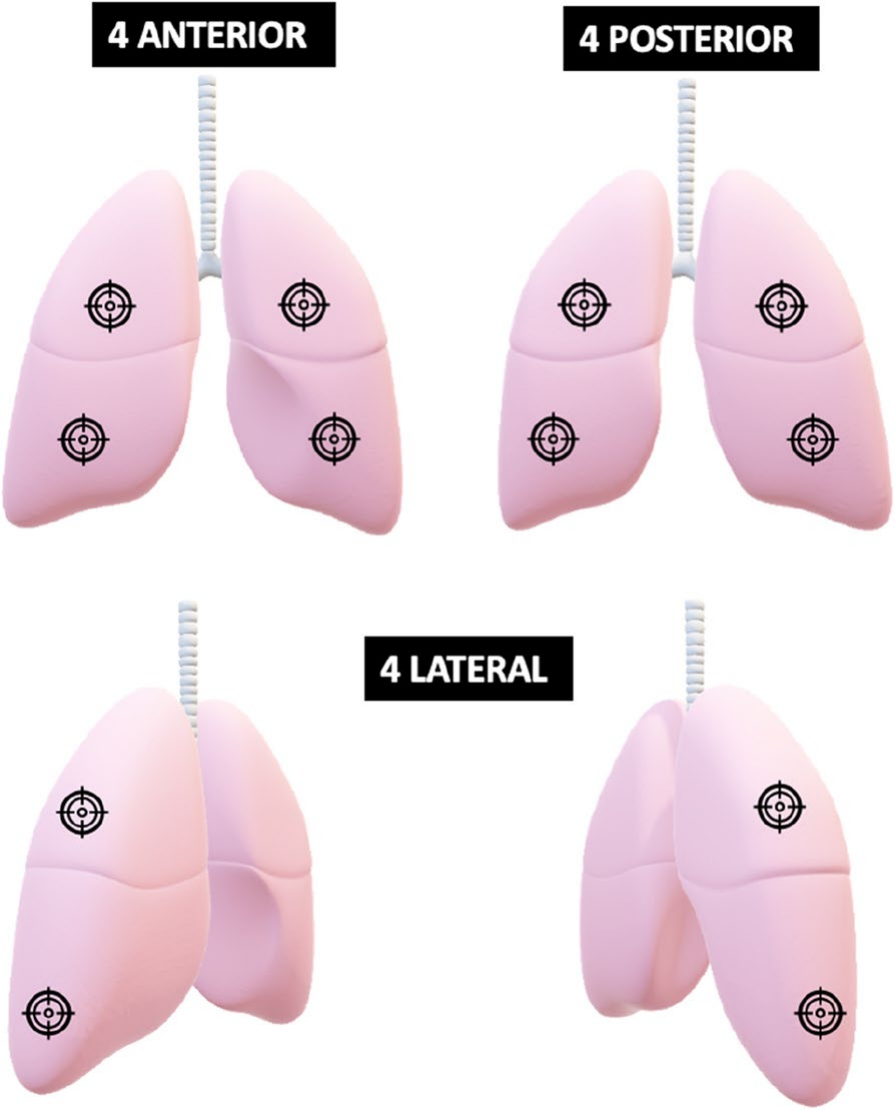
Lung points scanned by ultrasonography according to the study protocol

All patients have a *LUS* performed by a trained and study-certified ED physician following a standardized international evidence-based procedure for point-of-care lung ultrasound [27]. Six points per lung are scanned using a convex probe with depth adjusted to 8 cm from the pleural line to the bottom of the screen and with a correct gain (Fig. 2). The presence of pneumonia at LUS is defined as the presence of consolidation or hepatization over 1 cm or focal or unilateral B lines, following international evidence-based criteria [18, 27, 28]. These criteria were agreed upon by a group of eight LUS experts participating in the PLUS-IS-LESS study.

In case of high clinical probability of pneumonia (≥3 points on Van Vugt score) and/or presence of pneumonia on LUS, PCT is measured in the central laboratory of each participating hospitals. The turnaround time for PCT result is approximately 1 h. In patients with consolidation seen on LUS but a low PCT, a validated clinical severity score, (DS-CRB-65) [29] is used to detect patients with severe CAP who will need to receive antibiotics to ensure their safety (Fig. 2). The *DS-CRB-65 score* gives a point according to the following items: disease (D): presence of at least one of the following: congestive heart failure, cerebrovascular disease, chronic renal disease, chronic liver disease, or active malignancy; oxygen saturation (S) < 90%; confusion (C): altered mental status (Glasgow coma scale <15); respiratory rate (R) ≥ 30/min; blood pressure (B): systolic blood pressure < 90 mmHg or diastolic blood pressure ≤ 60 mmHg; and 65: age ≥ 65 years. The algorithm will recommend antibiotics in patients with a high severity score (≥3 points) regardless of the PCT result as it is associated with a 30-day 20% mortality risk. Of note, the clinical prediction and severity scores will be calculated automatically with the clinical patients’ data entered in the electronic case report form (eCRF) by the study team. The ED physicians are free to order other diagnostic tests.

##### Patients without features of pneumonia on LUS

In patients at low or intermediate clinical probability of CAP (estimated according to the clinical prediction score: Van Vugt score < 3 points), the probability of CAP with a normal LUS is low (expected pre-test probability of 40% with a NLR of 0.08, post-test probability of 6%) and the algorithm therefore does not recommend antibiotics. In patients with a high clinical probability of CAP (Van Vugt score ≥ 3 points), the post-test probability of pneumonia remains at an intermediate level even with a negative LUS (expected pre-test probability of 89% with a NLR of 0.08, post-test probability of 42%) and therefore PCT is recommended (Fig. 2). The algorithm will recommend antibiotics only in patients with elevated PCT using a standard cut-off of ≥ 0.25 μg/L [30].

##### Patients with features of pneumonia on LUS

PCT will be measured in all patients with LUS features of pneumonia to identify those in whom it is safe to withhold antibiotics (lower probability of bacterial or severe CAP in those with a low PCT value) [19, 20]. To ensure safety, a 28-day mortality risk will be calculated at the bed-side using the DS-CRB-65, and antibiotics will be prescribed if the score is ≥3 in patients with contradictory results (positive LUS and PCT <0.25 μg/L) [31].

##### Antibiotics recommendations

The PLUS electronic algorithm will recommend the prescription of antibiotics to the physician in charge through the eCRF. The choice, dosage, and duration of antibiotic treatment is left to the physician in charge.

##### Reassessment

PCT measurement will be repeated after 6 to 24 h in all admitted patients who did not receive antibiotics because of a low PCT value (< 0.25 μg/L). Antibiotics will be recommended in case of an elevated PCT (≥ 0.25 μg/L). PCT is also repeated after 6–24 h in patients in which the algorithm was overruled and antibiotics were prescribed, despite the recommendation. In case of low PCT value (< 0.25 μg/L), it will be recommended to stop antibiotics. If the physician in charge wants to prescribe antibiotics for the same infectious episode within the month of inclusion, PCT measurement is recommended and antibiotics prescription in case of elevated PCT.

Patients discharged home without antibiotics have an evaluation by phone after 48 h. In case of worsening symptoms (dyspnea, shivers, and/or fever) at 48 h, patients will be asked to come back to the ED for a medical evaluation and PCT measurement. Antibiotics will be prescribed in case of elevated PCT. In case of worsening disease within 28 days of enrolment in patients who did not receive antibiotics, patients will be invited to present to the ED where reassessment will be done, and PCT will also be repeated.

### Control group

The comparator will be routine care. Physicians working in an ED during the control period will manage the patients as they usually do. Physicians are provided with the guidance for CAP from the Swiss Society of Infectious Diseases (SSI) to standardized practices [26]. However, decisions on antibiotics and on the use of diagnostic tests are left to the physicians.

### Training

Before starting the study, ED physicians of all participating centers will receive a 1-h training on the epidemiology and management of CAP in Switzerland based on Swiss guidelines [27] as well as explanations of the rationale of the study.

*In the month prior to the intervention period*, the medical supervisors (senior registrars and senior physicians) of the respective ED will attend a 1-h presentation on the scientific data behind the algorithm. They will perform a 2-h e-learning training on LUS available on the learning platform “PocUS Academy” [32] offering structured teaching on ultrasonography, certified by the Swiss Society of Ultrasonography in Medicine. The respective supervisors will also attend a 1-h theoretical and 2-h practical training (1-h training on image acquisition on healthy volunteers and 1-h training on pneumonia diagnosis on patients admitted with pneumonia) led by an experienced instructor in LUS. This will be based on international recommendations [27, 28] and on the POCUS course of the Swiss Society of Ultrasonography (SGUM). Competencies in LUS will be assessed by an online quiz and a practical test with LUS image acquisition: physicians with previous expertise in LUS will record two examinations for review, while those without prior expertise will record five examinations, as suggested in the literature [33]. This assessment is based on the lung-ultrasound objective structured assessment of technical skills (LUS-OSAUS) [34]. Only physicians who achieved 80% of good quality image acquisition and 80% of correct answers in the online quiz will be allowed to perform LUS during the study. If quality objectives are not achieved, an extra one-to-one 1- to 2-h practical supervision will be offered. If the second test evaluation does not reach learning objective, the physician will not perform LUS in the study. If the objectives of the online quiz are not achieved, the 2-h e-learning will be repeated. If the second test evaluation does not reach learning objective, the physician will not perform LUS in the study.

*During the month after rolling into the intervention*, all recorded LUS (around seven per center) will be reviewed for quality and interpretation by a LUS expert with direct feedback to the physicians. If the quality or interpretation of LUS is insufficient, an extra one-to-one 1- to 2-h practical supervision will be offered to the concerned physicians.

### Endpoints

The primary safety endpoint is the proportion or patients with clinical failure by day 28. Clinical failure is defined as death from any cause, secondary intensive care unit (ICU) admission for any cause, secondary admission to hospital (excluding elective admission), hospital re-admission after index hospital discharge (excluding elective admission), or local complications due to the LRTI (empyema, lung abscess).

The primary efficacy endpoint is the proportion of patients prescribed an antibiotic by day 28. Table 4 summarizes the secondary and exploratory endpoints.

**Table 4:** Study endpoints

Primary safety endpoint	
Proportion of patients with clinical failure by day 28, defined as: death from any cause, or secondary ICU admission for any cause, or secondary admission to hospital (excluding elective admission), or hospital re-admission after index hospital discharge (excluding elective admission), or ­ local complications due to the LRTI (empyema, lung abscess)	
Primary efficacy endpoint	
Proportion of patients prescribed an antibiotic by day 28.	
Secondary endpoints	
Number of points on the CAP symptom questionnaire as a surrogate marker of quality of life on days 7, 28, and 90 in each study group.	
Duration of ED stay in each study group.	
Rate and duration of hospitalization in each study group	
Proportion of patients prescribed an antibiotic for a new respiratory infection in each study group between days 28 and 90.	
Proportion of patients with antibiotic-related side effects and *C. difficile* infections in each study group.	
Identification of barriers and facilitators to the implementation of the algorithm with patients and ED physicians involved in the study.	
Quality-adjusted life years (QALYs), derived from responses to the EQ-5D questionnaire, in each group	
Measurement of the incremental cost-effectiveness ratio (ICER) of the intervention as compared to usual care.	
Advanced automatic LUS image analysis method using machine learning to assist in LUS diagnosis and risk stratification.	
Proportion of physician reaching proficiency in LUS image/video acquisition and interpretation after the training module.	
Sensitivity, specificity, and area under the ROC of physician “gestalt” and Van Vugt prediction score for CAP diagnosis.	
Exploratory endpoints	
Proportion of LRTIs patients with a documented bacterial, viral, and mixed infection.	
Nasopharyngeal microbiome in adult Swiss patients with LRTIs.	
Urinary metabolite changes during antibiotic treatment.	
Presence and expression levels of new generation host biomarkers and transcriptome signature according to etiology and severity of the LRTI	

## Methods: Assignment of interventions

### Randomization, allocation, and timeline

**Fig. 3:**
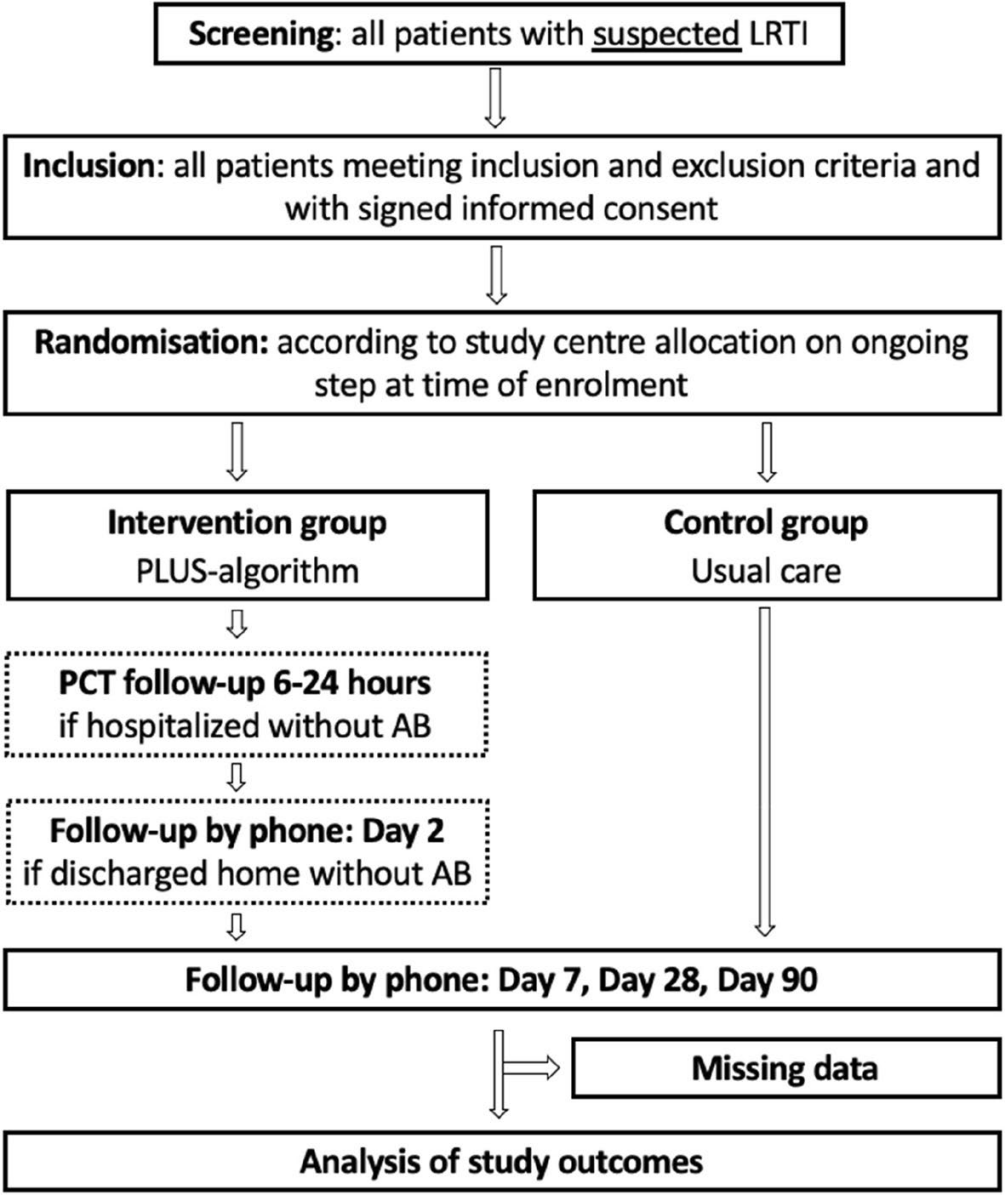
CONSORT flow diagram of the study. LRTI lower respiratory tract infection, PCT procalcitonin, AB antibiotic

The unit of randomization will be the nine centers, representing the clusters. In accordance with the stepped wedge cluster randomization design, after a common baseline 10-week-period of usual care (all patients included in the control group), one center will switch to the intervention phase (patients included in the intervention group) at each pre-determined time period (every 10 weeks, which corresponds to a step). All study centers will be in the intervention period for the last 10 weeks of the study (Table 5). The total duration of the study will be 113 weeks, including the follow-ups. The sequence of rolling into intervention (from control period to intervention period) was randomly generated between study centers using the web-based randomizing program: random.org. The nature of the study intervention precludes randomization at the patients’ level. Indeed, the intervention is targeting the decision-making process of the clinician and might contaminate the usual care group in case of patients’ randomization. Figure 3 represents the CONSORT flow diagram of the PLUS-IS-LESS study following SPIRIT guidance.

**Table 5:**
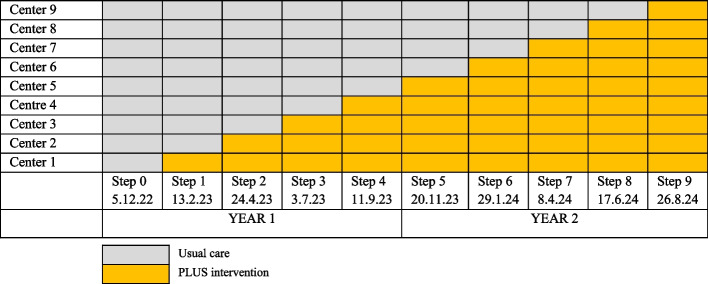
Stepped wedge roll out of the PLUS intervention by study center over time. Center 1: Cantonal Hospital of St Gallen, center 2: University Hospital of Lausanne, center 3: Hospital of Neuchâtel, center 4: University Hospital of Basel, Center 5: Cantonal Hospital of Lucerne, center 6 : Intercantonal Hospital of Broye, center 7: Hospital Riviera-Chablais, center 8: Cantonal Hospital of Baden, center 9: Cantonal Hospital Baselland & St Clarasspital Basel

### Blinding

Centers are randomly allocated to one cluster with pre-determined dates to switch from usual care (control group) to the intervention (intervention group). The assigned cluster was disclosed to the study centers in the month before initiating the study, as blinding is not feasible in this open-label trial. In this context, data analysts are not blinded either.

## Methods: Data collection, management, and analysis

### Data collection

Table 6 summarizes the schedule of enrolment, intervention, and assessments.

**Table 6:**
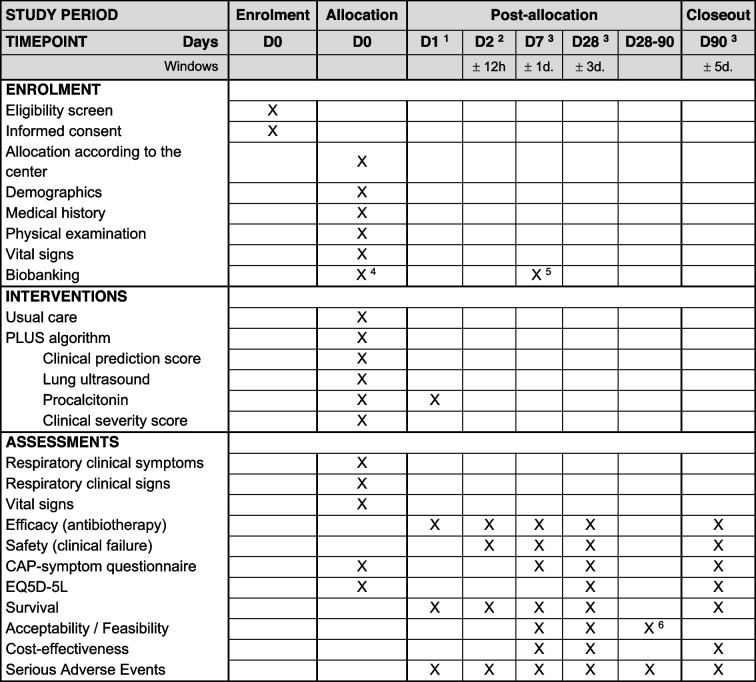
Schedule of enrolment, intervention, and assessments (as per SPIRIT [22])

^1^PCT will be repeated after 6 to 24 hours in all admitted patients who did not receive antibiotics because of a low PCT value and in those in whom the algorithm was overruled

^2^In the subgroup of patients discharged home without antibiotics: phone evaluation after 48 hours

^3^Study D7, D28 and D90 are done by phone by the study team

^4^Biobanking of plasma, whole blood, paxgene tubes, urine, nasopharyngeal and oral swabs and sputa

^5^Urine biobanking (urine metabolites) in a subgroup of patients

^6^Optional interview for qualitative assessment of acceptability of the algorithm in a subgroup of patients

### At enrolment

Study data will be collected (from the patient and/or the medical file) by the study team in an eCRF developed on the electronic data collection (EDC) system REDCap®, hosted at Lausanne University Hospital [35, 36]. Data collected at inclusion includes demographic characteristics, comorbidities, symptoms and their duration, vitals and clinical signs, diagnostic tests required by the ED physician for LRTI management of the included patients (chest X-ray, CT-scan, LUS, CRP, PCT, white blood cell count, blood chemistry, blood gas, microbiology tests), destination on ED discharge, and antibiotic prescription. During the intervention period, the data collection will also include PCT results and LUS interpretation. Furthermore, physicians will record all their LUS images and videos following the aforementioned procedure in a secured drive.

A collection of biological samples (blood, respiratory, and urine) is performed parallel to the intervention trial as a study data-linked biobank. This includes a collection of nasopharyngeal and oral swabs, sputa if available, whole blood, plasma, PAXgene™ blood RNA system tubes, and urine samples at inclusion in all study centers. Furthermore, urine samples are collected on day 7 in the University Hospital of Lausanne and the Cantonal Hospital of St Gallen.

### During follow-up

Study data will be collected by phone on days 7, 28, and 90. The study team will perform the follow-up phone calls. If the patient is admitted on the day of follow-up, the study team will interview the patient. They will also use the patient’s medical file if relevant. Data collected at follow-up includes mortality, hospitalization, use of oxygen therapy or non-invasive ventilation, presence of local complication of pneumonia, use of antibiotics, health care resources, LRTI-related symptoms, and evaluation of quality of life.

### Data management

Study data will be collected via an electronic case report form (eCRF) and managed using REDCap®. Trial and participants’ data will be handled with uttermost discretion and is only accessible to authorized personnel who require the data to fulfil their duties within the scope of the study. On the CRFs and other study-specific documents, participants are only identified by a unique participant number (3 to 4 letters for the site followed by 3 digits for the patient). Coding is generated by using the eCRF software. Biological material in this study is not identified by participant name but by a unique participant number. Biological material is appropriately stored in a restricted area only accessible to the authorized personnel.

Each recording and transcript collected during the qualitative interviews will be identified by the patient’s or physician’s unique participant number. To further ensure confidentiality, transcripts will be coded for any name or place that could allow to identify a participant.

### Quality management and monitoring

#### Monitoring

The monitoring activities are coordinated by the Clinical Research Center (CRC) Lausanne. The Clinical Trial Center Zurich (members of the Swiss Clinical Trial Organization (SCTO) and CTUs Network) is contracted by Lausanne to perform monitoring activities at some local sites.

A risk-adapted monitoring strategy were developed according to the SCTO guidelines (Guidelines for Risk-Based Monitoring, version 3.0). The monitoring strategy (nature and extent of the monitoring activities) were described in a monitoring plan. Site initiation visits were conducted on each site before the start of the trial. Then interim monitoring visits (6 per site) will be performed on site and focusing mainly on safety and patient eligibility. A closeout monitoring visit per study site will be performed at the end of the trial to ensure all pending actions are resolved and study documentation is ready for archival.

#### Quality controls

The quality controls include the evaluation of study center compliance to the protocol. Non-adhesion to the algorithm will be escalated to the sponsor as a study deviation triggering corrective action from the investigators. Furthermore, non-adherence to the algorithm recommendation will trigger automatic alerts sent to the sponsor for direct feedback to the clinician in charge. There will be a monthly follow-up of deviation from the research proposal. This includes an implementation evaluation of the intervention to detect problems, which may be corrected.

#### Safety monitoring

In our study, an adverse event (AE) is defined as any untoward medical occurrence in a participant which does not necessarily have a causal relationship with the trial procedure. A serious adverse event (SAE) is defined as any untoward medical occurrence that results in death or is life-threatening, requires in-patient hospitalization or prolongation of existing hospitalization, results in persistent or significant disability or incapacity, or causes a congenital anomaly or birth defect. Occurrence of SAEs from the first participant visit until the last follow-up phone will be actively sought. All SAEs occurring during the study will be documented in the eCRF and followed until resolution. SAEs identified as related to the intervention will be notified to the Ethics Committee according to regulatory requirements, as well as immediate safety and protective measures that should be taken during the conduct of the study.

#### Protocol amendments

The Sponsor is authorized to amend the protocol. All important protocol modifications will be first discussed within the Steering committee and then communicated to the relevant parties (local investigators, EC, trial registry) by the Sponsor. Substantial amendments will only be implemented after approval by the EC, whereas non-substantial amendments are communicated by the Sponsor to the EC within the annual safety report. Under emergency circumstances, deviations from the protocol to protect the rights, safety, and well-being of patients may proceed without prior approval of the EC. Such deviations shall be documented and reported by the Sponsor to the EC as soon as possible. Amended protocols will be sent to the study sites for filling in the Investigator Site File, and training on new documents will be documented on site.

### Analyses

#### Sample size

The study is designed to demonstrate non-inferiority of the intervention in term of clinical failure occurrence and superiority in term of reducing antibiotic prescription. We plan to include 1530 patients in 9 study centers. Every study center will recruit 170 patients over about 2 years.

According to a stepped-wedge cluster-randomized design with a control period plus nine switch periods (steps) and the same number of clusters (one switch to intervention at each step), a mean number of 15 patients per unit and per time period will guarantee a 80% power (one-sided type I error *α* = 0.05) to prove non-inferiority regarding clinical failure (safety), if the proportion of clinical failure is 20% in both groups, with a non-inferiority margin of 10%, and assuming an intra-cluster correlation (ICC) of 0.2 and a coefficient of variation (cv) of the number of patients across units of 0.3. With 15 patients per unit and per time period, we would obtain a total sample size of 1350 (15 × 9 × (9+1)) patients. Further considering a 10% of lost to follow-up, we anticipate 15/0.9 = 17 patients per unit and per time period, leading to our final sample size of 1530 (17 × 9 × (9+1)) patients.

In our calculation, the ICC of 0.2 is based on results of a previous trial, the UltraPro trial [37]. The coefficient of variation of 0.3 in the number of patients between units is intended to allow for some variability between centers in the number of patients recruited. With an average number of 15 patients per cluster and per step, this corresponds to admitting that 95% of the clusters will recruit between 6 and 24 patients per step (between 60 and 240 in total). Correcting for 10% loss to follow-up, the same range will be between 67 and 267.

Of note, the 10% non-inferiority margin is supported by US FDA recommendations for anti-infectious trials assessing clinical success of a new treatment [38]. Sample size calculation with a 5% margin would lead to 5100 patients jeopardizing the feasibility of the trial.

This sample size in the framework of a stepped-wedge design guarantees a power of more than 90% to prove superiority regarding antibiotics prescription (efficacy), if the proportion of antibiotics prescribed is 0.65 in the control group and 0.5 in the intervention group (*α* = 0.05).

The sample size was calculated with R software, based on the manuscripts by Hemming et al. [39] and Harrison et al. [40].

### Statistical analyses

#### Datasets to be analysed, analysis populations

All analyses will be carried out in the primary analysis population, which includes all enrolled patients following an intention-to-treat principle. Any missing data at the enrolment visit due to incomplete documentation of the pre-assigned components of the algorithm will lead to the patient being assigned to the intention-to-treat analysis. Patients who are lost to follow-up will be replaced by additional patients’ inclusion as mentioned in the sample size (10% additional patients to compensate for loss to follow-up). Patients lost to follow-up will not be included in the analysis (complete case analysis). The primary efficacy and safety analyses will be repeated on the per-protocol population. The per-protocol population includes all patients who received all components of the PLUS algorithm without overruling on admission and 6–24 h later.

Stratified analyses will be done for severe LRTIs and non-severe LRTIs based on CRB-65 and PSI scores.

A sensitivity analysis will include patients who received a component of the intervention (PCT or LUS) while in the “usual care group” to evaluate if there is still an impact of the intervention when these tests are part of the usual care.

Additional analysis adjusting for patient level confounding factors (age, asthma, chronic obstructive pulmonary disease, CRB-65, and PSI score) in the intention-to-treat analysis will be done for both co-primary endpoints.

We will develop a detailed data analysis plan for secondary, subgroup, stratified, and sensitivity analyses. All analyses will be done with R Statistical Software.

#### Primary analysis

##### Primary safety and efficacy endpoints

Mixed effect generalized linear (logistic) models will be implemented in order to estimate the effect of intervention on safety (clinical failure) and efficacy (antibiotic prescription). The models will contain a random cluster level effect and a period specific fixed effect. Both intervention effects will be expressed in an odds ratio (OR) scale. A lower one-sided 95% confidence interval will be used in order to test effect significance for the safety outcome, reached when the upper bound of the interval is smaller than the non-inferiority margin (translated into the OR scale); a two-sided 95% confidence interval will be used to test effect significance for the efficacy outcome, reached when the interval does not contain the value of one.

#### Secondary analyses

*The effect on bothersomeness of community-acquired pneumonia-related symptoms* on day 7, day 28, and day 90 will be evaluated by comparing the number of points on the CAP symptom questionnaire between study groups by linear mixed effect models containing a random cluster level effect and a period-specific fixed effect. The effect on the *duration of ED stay will be evaluated* using survival models including frailty term for the cluster effect (Coxmodel).

##### Acceptability and feasibility of the intervention

We will conduct semi-structured interviews with patients (approximately 40 patients at 4 sites and/or until data saturation is reached) and ED physicians (1–2 per site and/or until data saturation is reached) using a predefined interview guide. The four sites will be differentiated according to university/peripheral, urban or rural, French-speaking, or German-speaking contexts. These are the University Hospital of Lausanne, Intercantonal Hospital of Broye, University Hospital of Basel, and Cantonal Hospital of Baden. Patients’ selection will be stratified according to age, sex, nationality, and level of education. To ensure that they are representative of the population with LRTI in EDs, we will focus on a population aged 59 years and over (median age of 73 years, based on the ProHOSP randomized controlled trial) [20]. Half of the patients will be part of the control group (managed as usual), and half will be part of the intervention group (managed with the PLUS intervention) to highlight the differences in perception between usual care and a management with new diagnostic tools. All interviews will be audio-recorded and transcribed verbatim.

Using a pre-defined interview guide and a content analysis approach, data will be coded using a qualitative data analysis software and analyzed in regard to the identification of barriers and facilitators to the implementation.

##### Cost-effectiveness of the intervention

We will first calculate the within-trial technical Incremental Cost-Effectiveness Ratio (ICER), expressed in Swiss francs (CHF) per percentage point reduction in antibiotic prescription using the PLUS clinical management algorithm as compared to usual care. This will be complemented by a condition-specific quality-of-life ICER, expressed in CHF per point reduction in the CAP-symptom questionnaire. Then, we will use responses to the EQ-5D questionnaire to derive quality-adjusted life years (QALYs) using an area under the curve approach and an appropriate value set (e.g., German), which will allow us to express the cost-utility of the intervention in CHF per QALY gained. Finally, to incorporate the potential wider benefits of a reduction in antibiotics prescriptions, we will design a long-term economic model to calculate the social burden attributable to anti-microbial resistance [41]. An epidemiological model to measure the spread of resistant strains will be developed and the cost per case of individuals affected by such an infection will be estimated [42].

##### Deep learning for clinical decision support

We will explore the ability of deep learning to identify the diagnostic (including etiology) and prognostic patterns of pneumonia from a flexible combination of clinical data and LUS images. Adapting the approach from Trottet et al. [43], we will create a multimodal, multi-task modular decision support network (MoDN). MoDN is *multimodal*, in that it can input various data types (e.g., LUS images, tabular data for age, PCT, etc.). MoDN is *multi-task*, in that it can predict multiple targets at once (e.g., pneumonia, bacterial pneumonia, severity score, etc.). MoDN is *modular,* in that it comprises a flexible series of neural network encoder modules (specific to each input) and decoder modules (specific to each task).

This approach has the advantage of being composable at the bedside, where the clinician may select any combination or number of inputs according to their availability and receive a statistically calibrated prediction.

We will compare this to several clinical baselines such as the PLUS algorithm and LUS expert interpretation. LUS expert interpretations of each image will either be summarized by an existing diagnostic score or handled by a newly developed machine learning model (such as a dense network or random forest).

##### Optimization of the clinical management algorithm

We will evaluate the PLUS algorithm and the multimodal deep learning algorithm and apply interpretability techniques to identify the most informative features for the outcome. MoDN is inherently interpretable and feature importance of each input can be directly extracted from the algorithm. An optimal order and combination of inputs will be explored. The PLUS algorithm will also be assessed, and various modelling approaches will be tested (random forest, logistic regression, dense networks etc.), with several feature selection methods (forward and backward feature selection, lasso etc.) to identify the minimal number of inputs required and better understand the importance of each element.

### Data safety monitoring board

The presence of such board was deemed unnecessary in view of the low risk associated with this study.

### Time plan for the study

The planned overall study duration is 2 years and 3 months (113 weeks), including the recruitment period and study duration for each patient. Patient recruitment began in December 2022, the last-Participant-Out will be in March 2025.

## Discussion

This clinical trial evaluates the safety and efficacy of a clinical algorithm including a clinical prediction score, PCT and LUS, to decide on antibiotic prescription in patients presenting with LRTIs to EDs. The clinical algorithm was built to optimize diagnostic performance to ensure safety and to maximize physician adherence to the algorithm’s recommendation.

The study population is selected using meaningful, reproducible, and nonrestrictive inclusion criteria, as well as only few exclusion criteria. In this pragmatic trial, the study population will be representative of the targeted real-life population. However, patients with severe underlying lung disease, severe immunosuppression, or clinical instability requiring ICU are excluded to ensure safety.

The stepped-wedge cluster randomized design of the trial overcomes many challenges faced during intervention studies targeting physicians and including patients with LRTIs. The cluster design prevents cross-contamination between study groups, as physicians are not exposed to the intervention during or before the control “usual care” period. Contamination is an important issue in trials when evaluating new diagnostic interventions focusing on physicians and where individual patients are managed by the same physicians, but randomized to different groups/managements. It is highly likely that diagnostic tools used in the intervention group spill over to the control group. The result of contamination is that outcome differences between the treatment groups would be diluted, biasing the trial towards the null hypothesis.

The stepped-wedge cluster design reduces the disadvantages and limitations of a parallel cluster randomized trial. It implies that all clusters (EDs) start in the control period. Then, the clusters switch to the intervention period at consecutive time points, where the time of the switch is randomized for every cluster. Eventually, all clusters will have switched from the control to the intervention period. The main advantage of this design is that the clusters act as their own controls because they are active both as control and intervention. Therefore, the intervention effect can be estimated from both between- and within-cluster comparisons. This results in more statistical power and smaller required sample sizes than in parallel cluster design [44].

The stepped-wedge design also decreases the risk that the characteristics of the study population differ between groups, as it may be the case in a parallel cluster design, where EDs may admit patients with different characteristics. In addition, this design allows to control for and estimate the effect of seasonality on outcomes, since each season of the year and each year (controlling for yearly variability in circulating respiratory pathogens) will be represented in usual care and interventions groups. Therefore, this stepped-wedge study design is a type of cluster randomized controlled trial well-suited to study acute LRTIs and evaluating algorithm-based treatment decisions. It also facilitates post-study implementation in case of positive results, as all centers are exposed to the intervention at the end of the study [45, 46].

This is a low-risk study for participants, comparable to standard of care, as it is based on previously validated tests. A potential risk is related to the inappropriate withdrawal of antibiotic treatment in patients with CAP which might be due to missing central infiltrates on LUS and to the non-perfect sensitivity of PCT. However, there are defined safety measures in the study design to diminish such risks: neither patients with severe symptoms, requiring intensive care, nor patients with severe chronic obstructive pulmonary disease (COPD) or severe immunodeficiency will be included. Moreover, procalcitonin-guided prescription in patients with LRTIs has been previously shown to be safe in patients with severe LRTIs [20] in ED and in a primary care settings [47, 48]. A clinical severity score will further ensure the safety of the intervention in those with discordant results between the components of the algorithm (LUS consolidation and low PCT). PCT will be repeated after 6–24 h in admitted patients who did not receive antibiotics. Patients discharged without antibiotics will benefit from a phone evaluation after 48 h.

In this pragmatic trial, we chose to compare our intervention to “usual care” instead of “standard of care” to confront it to real clinical practice. In order to increase homogeneity of the usual care, ED physicians of all centers will receive a training on the epidemiology and management of CAP in Switzerland based on Swiss guidelines [26].

We expect that there will be some overruling, when antibiotic prescription is not recommended. To decrease the risk of overruling, we presented the rationale behind the algorithm during the site initiation visits. In patients in whom the algorithm is overruled and antibiotics are prescribed in spite of the recommendation of the algorithm, PCT will be repeated after 6–24 h. In case of low PCT levels, it will be recommended to stop antibiotics. A close monitoring will be performed to rapidly detect and correct non-compliance problems.

The PLUS algorithm based on clinical scores and easy-to-use diagnostic tests has the potential to improve the identification of patients with a LRTI who will benefit from antibiotics and reduce unnecessary antibiotic treatments. This diminished prescription of antibiotics will decrease side effects, cost, and resistance which are global health problems. The World Health Organization Trial Registration Data Set for PLUS-IS-LESS study is displayed in Table 7.

**Table 7:** World Health Organization Trial Registration Data Set for PLUS-IS-LESS study

Data category	Information
Primary Registry and Trial Identifying Number	www.ClinicalTrials.gov, NCT05463406
Date of Registration in Primary Registry	19 June 2022
Secondary Identifying Numbers	BASEC number 2022-00738
Trial Protocol version	Study protocol V3.0 dated 28.02.2023
Source(s) of Monetary or Material Support	Grant: SNSF 33IC30_201300
Primary Sponsor	CHUV
Secondary Sponsor(s)	Not applicable
Contact for Public Queries	NBB, noemie.boillat@chuv.ch
Contact for Scientific Queries	NBB, noemie.boillat@chuv.ch
Public Title	Procalcitonin and Lung Ultrasonography Guided Antibiotherapy in Emergency Departments, The PLUS-IS-LESS study
Scientific Title	Procalcitonin and Lung UltraSonography based antibiotherapy in patients with Lower rESpiratory tract infection in Swiss Emergency Departments: a pragmatic stepped-wedge cluster-randomized trial
Countries of Recruitment	Switzerland
Health Condition(s) or Problem(s) Studied	Lower respiratory tract infections
Intervention(s)	The PLUS clinical management algorithm: The PLUS algorithm starts with a validated pneumonia clinical prediction score (score of Van Vugt), followed by LUS. In case of positive results of any of these tests, PCT is measured to identify patients who will most likely benefit from antibiotics. A validated clinical severity score will ensure the safety of the intervention in those with discordant results (LUS consolidation and low PCT). Usual care: management as usual
Key Inclusion and Exclusion Criteria	Inclusion Criteria: • Patients aged 18 years or more • Acute LRTI (acute illness, less than 21 days, with at least one lower respiratory tract symptom, i.e. cough, sputum, dyspnea, chest pain and no alternative explanation) • At least one of the following clinical criteria: ◦ Focal abnormal auscultation (decreased breath sounds, crackles, bronchial breath sounds) ◦ Fever (documented temperature ≥ 38°C in the last 24 hours, including self-measured temperature ≥ 38°C) ◦ Tachypnea (respiratory rate ≥ 22/minute) ◦ Tachycardia (heart rate ≥ 100/minute) Exclusion Criteria : • Previous receipt of a quinolone, macrolide or ceftriaxone or, of more than one dose of any other antibiotic within 72h prior to enrolment (excepted prophylactic antibiotics or antibiotics given for urinary tract infection) • Previous acute care hospital stay in the last 14 days • Cystic fibrosis • Severe COPD (≥GOLD 3 or if not available, as a proxy: exacerbation treated with antibiotics during the last 6 months) • Severe immunodeficiency (drug-induced neutropenia with <500 neutrophils/mm3, HIV infection with CD4<200 cells/mm3, solid organ or bone marrow transplant recipient, prednisone ≥ 20mg/day for >28 days) • Initial admission of the patient in the intensive care unit • Microbiologically-documented SARS-CoV-2 (in the last 10 days) • Lack of decision-making capacity
Study Type	Type: Investigator-initiated, interventional, pragmatic study Allocation: Randomized Intervention model: Stepped-wedge rollout Masking: None (Open Label) Primary purpose: Diagnostic Phase: Phase IV
Date of First Enrollment	5 December 2022
Sample Size	1530 patients
Recruitment Status	Recruiting
Primary Outcome(s)	Safety outcome: Proportion of patients with clinical failure at day 28 (defined as a composite of any of the following: death or secondary ICU admission or secondary admission to hospital or hospital re-admission after index hospital discharge or complications due to the LRTI [empyema, lung abscess]) Efficacy outcome: Proportion of patients prescribed an antibiotic in each intervention group between enrolment and day 28
Key Secondary Outcomes	Quality of life measured with the community-acquired pneumonia symptom questionnaire on day 7, 28 and 90 Duration of ED stay in each study group Rate and duration of hospitalization in each study group Proportion of patients prescribed an antibiotic in each study group between enrolment and day 28. Proportion of patients prescribed an antibiotic for a new respiratory infection in each study group between day 28 and 90. Antibiotic side effects and C. difficile infection, from day 0 to 28 Acceptability and feasibility of the intervention through extensive identification of barriers and facilitators in patients and physicians conducting qualitative semi-structured interviews Quality-adjusted life years (QALYs), derived from responses to the EQ-5D-5L questionnaire, in each group Cost of the intervention as compared to usual care Advanced automatic LUS image analysis method using machine learning to assist in LUS diagnosis and risk stratification. Proportion of physician reaching proficiency in LUS image/video acquisition and interpretation after the training module. Sensitivity, specificity and area under the ROC of physician “gestalt” and Van Vugt prediction score for CAP diagnosis
Ethics Review	Approved on 29.11.2022 (BASEC number 2022-00738).
Completion date	March 2025

### Supplementary Information

**Additional file 1.** SPIRIT Checklist

## Data Availability

The participant information materials, informed consent form, the datasets analyzed during the current study, and statistical code will be available from the corresponding author on reasonable request, as is the full protocol. Furthermore, selected coded data and statistical code will be transferred on appropriate open data repositories (such as Zenodo or Figshare) to ensure data accessibility in the long-term, with the purpose of reproducibility of analyses.

## Abbreviations

AB: Antibiotic
CAP: Community-acquired pneumonia
CHF: Swiss francs
EC: Ethics committee
eCRF: Electronic case report form
ED: Emergency department
ICER: Incremental cost-effectiveness ratio
LRTI: Lower respiratory tract infection
LUS: Lung ultrasound
LUS-OSAUS: Lung-ultrasound objective structured assessment of technical skills
PCT: Procalcitonin
QALYs: Quality-adjusted life years
SGUM: Swiss Society of Ultrasonography
TIDieR: Template for Intervention Description and Replication
